# Brewer’s Yeast for Pyogenic Infections

**Published:** 1905-03

**Authors:** 


### BREWER'S YEAST FOR PYOGENIC INFECTIONS.

It is stated that the workers in some breweries in France have for many years made use of brewer's yeast in the treatment of acne, furunculosis and malignant pustules. The first mention of this remedy in medical literature is by Mosse, in 1852. Brocq claims that he used yeast on himself in the treatment of recurrent malignant pustules, and was entirely cured, although he had tried many other remedies without obtaining relief. He reports in detail one other case of malignant pustules, three cases of furunculosis and one case of acne, all of which it is alleged were cured with brewer's yeast. Lassar used it in the treatment of furunculosis complicating diabetes, and reports equally satisfactory results. Many other reports may be found in the recent literature, nearly all of which speak highly of this simple remedy. The best results are obtained with fresh brewer's yeast (*Saccharomyces cerevisiæ,*) although baker's yeast has also been used. It is taken internally in doses of from a teaspoonful to a dessertspoonful dissolved in a glass of water. This may be drunk during or at the beginning of each meal, three times a day. It is important that the yeast should be fresh, because slight digestive disturbances, and occasionally diarrhea, are produced even by perfectly fresh yeast.

Recently yeast has been locally applied in the treatment of gonorrheal affections in women. It has been used in the form of balls which are introduced into the vagina and also in the form of suppositories which are introduced into the cervix. Some very satisfactory results have been reported from this treatment by Abraham[1] and others. Cronbach,[2] however, does not think that it has any great advantage over the older treatment with antiseptics.

The exact mode of action of this remedy is not clearly understood, although several attempts have been made to throw light on this question by test-tube and animal experiments. Sergent inoculated rabbits with staphylococcus aureus by rubbing the cultures into the shaved skin. This inoculation produced many small furuncles, which healed rapidly under the administration of yeast. The controls always required from four to five days longer for recovery. When large doses of staphylococcus were injected under the skin the rabbits invariably died from the infection. McNair Scott could not notice any difference in favor of the treated rabbits when observing a large number of animals that had been inoculated with staphylococcus. Turro, Tarruella and Presta[3] inoculated six rabbits under the skin of the ear with several drops of a virulent streptococcus. Four of these rabbits were treated with yeast by subcutaneous injections, and the other two got no treatment. All of the animals developed erysipelas of the ear, but those that were treated soon improved and were practically well on the seventh day, while both controls were still becoming worse. One of the controls showed signs of improvement on the tenth day, and made a slow recovery, but the other died of a generalized infection on the eleventh day. Somewhat similar results were obtained from inoculation with *staphylococcus pyogenes aureus* and *albus*. These investigators, as well as Sergent, were able to

[1]Centralbl. f. Gynäkologie, 1901, xxviii, p 249. Abstracted in *The Journal*, April 2, 1904, p. 931.
[2]Ibid, 1904, xxviii, p. 1356. Abstracted in *The Journal*, Dec. 31, 1904, p. 2054.
Centralbl. f. Bakt., 1903, vol. xxxiv, p. 22.

produce a fair degree of immunity in rabbits, to the pyogenic micrococci, by treating the animals with yeast for a number of days before the inoculation. This immunity, however, was soon lost after discontinuation of the treatment. The serum of animals thus treated acquired high agglutinative properties for streptococci and staphylococci, but was not bactericidal. Extract of yeast cells has not given as satisfactory results as the whole yeast. It would seem from these reports that the administration of yeast has a favorable influence over many mild forms of pyogenic infection, and as it is a very simple and harmless remedy its use can be safely recommended.—*Jour. of the Amer. Med. Association.*

